# Knee Buckling as an Atypical Adverse Effect of Clozapine: A Case Report

**DOI:** 10.7759/cureus.55865

**Published:** 2024-03-09

**Authors:** Javeria Sahib Din, Ernesto Navarro Garcia, Hiba Al-Rubaye, Carlos Julian

**Affiliations:** 1 Psychiatry, Kings County Hospital Center, New York, USA; 2 Nanotechnology, University of Central Florida, Orlando, USA; 3 Physiology and Neuroscience, St George's University, St. George's, GRD; 4 Physiology and Neuroscience, St. George's University School of Medicine, St. George's, GRD

**Keywords:** knee buckling, therapeutic range, drug-related side effects and adverse reactions, negative myoclonus, clozapine side effects

## Abstract

Clozapine has become a widely popular and effective medication in the treatment of refractory schizophrenia and refractory bipolar disorder. Although the use of clozapine proves to be an effective resort, it has to be closely monitored due to its narrow therapeutic range and multiple dangerous adverse effects. In rare cases, clozapine has been known to cause an antagonistic myoclonic jerk that leads to knee buckling. Here, we present the case of a 29-year-old female who is being treated for schizoaffective disorder, bipolar, manic type, who reported two instances of knee buckling associated with falls while taking clozapine.

## Introduction

Clozapine is an atypical antipsychotic that is usually reserved for refractory cases [1]. The mechanism of action of clozapine is not fully understood. However, data suggests that it primarily acts via antagonism of dopamine D2 and serotonin 2A receptors [2] and by displaying some anticholinergic, antihistaminic, and anti-adrenergic effects [3]. Such mechanisms of action are largely responsible for the plethora of side effects and the need for close monitoring of serum levels in all patients who take this medication regularly. Among the many side effects, sedation, drooling, constipation, agranulocytosis, cardiomyopathy, seizures, and extrapyramidal effects (EPS) are among the most commonly reported side effects [4]. Regarding EPS, the commonly reported adverse effects of clozapine include tremors, muscle rigidity, and tardive dyskinesia [5]. In exceptionally rare circumstances, a negative myoclonic reaction has occurred, causing patients to feel a knee-buckling sensation that sometimes leads to falls. These negative myoclonic reactions have also been reported to occur with gabapentin and pregabalin [6]. The occurrence of a negative myoclonic reaction leading to knee buckling due to clozapine has been scarcely reported [7,8]. Many mechanisms have been deemed to be involved in the occurrence of this reaction, namely, problems at the neuromuscular junction and abnormal firing from cortical and subcortical regions [9], and inhibition of the primary sensorimotor cortex of the affected limbs. This is considered as a particular epileptic syndrome that appears in benign childhood epilepsy with centrotemporal spikes (BECTS) [10]. The importance of acknowledging this potential side effect is important due to the associated risk of falls that may follow and potential injuries that may occur as a consequence, particularly in the elderly.

## Case presentation

A 29-year-old female undergoing treatment for schizoaffective disorder, bipolar, manic type, was initiated on clozapine following multiple failed trials of haloperidol (5 mg BID, discontinued due to excessive sedation), risperidone (6 mg daily, discontinued due to failure of symptomatic improvement), and Olanzapine (20 mg, discontinued due to comorbid obesity). Due to the refractory nature of the presentation, other pharmaceutical options were considered. At the time, the patient was being cared for by a different treating psychiatrist on a different floor within the same institution. When the patient was transferred to our unit for continuation of care and discharge assessment, her current prescribed dosage was 425 mg of clozapine daily with close monitoring of therapeutic serum levels shown in Table 1.

**Table 1:** Serum levels of clozapine during the reported side effect of knee buckling and the associated dosage. Table created by author ENG and HA.

Date of clozapine serum level check	Serum levels of clozapine in ng/mL	Clozapine dosage in mg
05/02/23	656	450
05/17/23	1052	450
05/31/23	809	450
06/13/23	815	450
07/11/23	1083	425

During this time, on 6/28/23, the patient sustained a fall due to reported "knee buckling,” causing her gait to become unstable. The patient reported a subjective feeling of “locking knees” while ambulating which she attributed to her loss of footing and subsequent fall. The fall was associated with knee tenderness and a mild abrasion. At the time of the fall, no loss of consciousness (LOC) or seizure activities preceding the fall were reported and physical examination showed no focal neurological findings. Following this event, the medication dose was reduced to 425 mg daily by her previous physician. On 7/10/23, the patient reported having another episode where both knees buckled causing a fall without major consequences. Again, no LOC or seizure activity was reported at this time and no focal neurological findings were present. At this point, the patient was transferred to our unit within the same facility, and the transfer of care to our floor was completed. The clozapine dose was reduced to 350 mg daily on 7/14/23 by her new treating psychiatrist and the therapeutic levels have remained stable since as shown in Table 2.

**Table 2:** Serum levels of clozapine after dosage reduction and the associated dosage. Table created by author ENG and HA.

Date of clozapine serum level check after dose adjustment	Serum levels of clozapine in ng/mL	Clozapine dosage in mg
08/23/23	604	350
09/23/23	621	350
10/23/23	678	350

As of today, no more episodes of falls have been reported, and the patient has not complained of knee buckling. The patient is noted to walk with a normal gait, proper posture, and adequate pace. She is able to complete her activities of daily living independently and can tour different areas of the unit without complaints. Current plans for this patient include continuous monitoring of her clozapine serum levels, participation in group and individual therapy, as well as continuous assessments to determine if the patient can safely return to the community. 

## Discussion

The majority of side effects caused by clozapine are well-known [4,5]. Due to these potential adverse effects, constant blood work monitoring is performed to ensure appropriate therapeutic levels are attained and to monitor the potential for cardiomyopathy and agranulocytosis. The knee buckling episodes associated with clozapine are rare [7,8], and the resolution of the adverse effects had mixed results.

In prior case reports, the negative myoclonic jerk was reported as an adverse effect that started with sudden dose changes of clozapine. For some of the patients in prior reports, the resolution of the negative myoclonus was partial or full with the reduction of clozapine dosages and the addition of other medications, such as valproic acid [7] which could potentially explain the mixed results in the resolution of the adverse effect in some patients. In particular, these older reports argue that the occurrence of the negative myoclonus is due to sudden changes in clozapine dosages, even if the changes go from a high to a low dose. Furthermore, they suggest that the treatment of myoclonus may be dualistic: one, reducing the dosage of clozapine, and two, adding an anticonvulsant as an adjuvant. However, they fail to mention the serum levels of clozapine at the time of the reported events. Although they mention continuous blood work monitoring, no serum level values were provided at the time of occurrence, nor at the time of resolution.

In our case, the patient complained of a knee-buckling sensation in both instances leading to her fall. The falls were witnessed in both episodes, and no previous seizure-like activity, stroke, or LOC was witnessed prior to the fall. At the time, her therapeutic levels were above the desired therapeutic range of 250 - 550ng/mL [11] (older studies suggest up to 1000ng/mL [12]) as shown in Table 1. However, in cases of refractory schizophrenia, a range above 350ng/mL may be the most optimal for an adequate response. Nonetheless, the patient's serum levels at the time of the reported knee buckling were much higher than the current recommended research data suggests. During these times, the patient reported two episodes where they described a knee-buckling sensation which was then followed by a fall that did not result in further complications. With the appropriate reduction of clozapine dosage, the therapeutic levels were reduced and maintained at lower ranges between 600 and 680ng/mL. Since the serum correction, the patient has not complained of similar knee buckling episodes, and her psychiatric conditions have remained stable. The patient has been able to ambulate with proper gait, pace, and biomechanical motions, and no falls have been witnessed. These findings suggest that appropriate maintenance of clozapine at the therapeutic range was sufficient to eliminate the adverse reaction completely.

The first takeaway from our case is that high serum levels of clozapine are likely to be related to the occurrence of negative myoclonus rather than the dose of clozapine itself. This claim is based on the fact that our patient underwent a significant dose change from 425mg to 350mg that resulted in full resolution of the adverse effect at a relatively high dose, whereas in previous reports, the negative myoclonus occurred even in lower dosages. The second important result of our case is that the reduction of clozapine serum levels via dose adjustment may be the only necessary step to achieve a complete resolution of the negative myoclonus. This second result is particularly important because it avoids the need to add other pharmaceuticals to an already complex regimen of medications, as is the case in most severe psychiatric conditions. This avoidance of other medications eliminates more unwanted side effects and potential drug-to-drug interactions. Furthermore, the addition of other medications to treat the negative myoclonus may play a role in the remission of the side effects. Without knowing the serum levels of clozapine, it is not certain if the adverse effect in prior cases was resolved due to the dose reduction of clozapine, the addition of anticonvulsants, or both. As a result of this, we propose that in cases where negative myoclonus appears in patients taking clozapine, careful monitoring of serum levels is done first to reduce it to appropriate ranges.

Limitations and considerations

The case report uses as much information as possible with the little subjective information received from the patient at the time of the reported side effects. Also, the patient had two different treating physicians during this time, which explains why the initial dosages and modifications are as such. Furthermore, our conclusion is based upon existing data from prior reports that reported mixed results with dosage changes and the addition of valproic acid. This is particularly important as valproic acid itself could play a role in the lack of resolution in previous cases. 

## Conclusions

This case highlights a rare but noteworthy side effect associated with clozapine, namely negative myoclonus otherwise known as knee buckling episodes which occurred when the serum therapeutic levels of clozapine exceeded the desired range. The prompt identification of this adverse reaction and subsequent adjustment of clozapine dosage led to a resolution of the patient's symptoms and the continuation of stable psychiatric progression. This underscores the importance of vigilant monitoring and individualized dosage management in patients undergoing clozapine treatment, particularly the elderly or patients placed at high risk for falls. The successful outcome, in this case, demonstrates that maintaining the medication within the therapeutic range can mitigate adverse effects, emphasizing the need for a personalized approach in the use of clozapine to optimize both efficacy and safety for individuals with psychiatric conditions.
